# Correction: Diamant et al. Effectiveness of Early Radical Cystectomy for High-Risk Non-Muscle Invasive Bladder Cancer. *Cancers* 2022, *14*, 3797

**DOI:** 10.3390/cancers14236001

**Published:** 2022-12-05

**Authors:** Elliott Diamant, Mathieu Roumiguié, Alexandre Ingels, Jérôme Parra, Dimitri Vordos, Anne-Sophie Bajeot, Emmanuel Chartier-Kastler, Michel Soulié, Alexandre de la Taille, Morgan Rouprêt, Thomas Seisen

**Affiliations:** 1 Sorbonne Université, Department of Urology, GRC n°5 Predictive Onco-Urology, AP-HP, Pitié-Salpêtrière Hospital, 75013 Paris, France; 2 Department of Urology, CHU-Institut Universitaire du Cancer-Oncopôle, 31000 Toulouse, France; 3 Department of Urology, University Hospital Henri Mondor, APHP, UPEC, 94000 Créteil, France

In the original article [1], there were mistakes in Figure 1, Figure 2, Figure 3, Figure 6, Figure 7 and Figure 8 as published. These survival curves are those with T0 set at the time of initial transurethral resection of the bladder tumor, while we chose to publish the results of survival analyses with T0 set at the time of early radical cystectomy (eRC) to report on the specific effect of this procedure for high-risk non-muscle invasive bladder cancer. All the results in the main text are correct with T0 set at the time of eRC, but the survival curves do not match these results. The corrected Figure 1, Figure 2, Figure 3, Figure 6, Figure 7 and Figure 8 appear below. The authors apologize for any inconvenience caused and state that the scientific conclusions are unaffected. The original article has been updated.

## Figures and Tables

**Figure 1:**
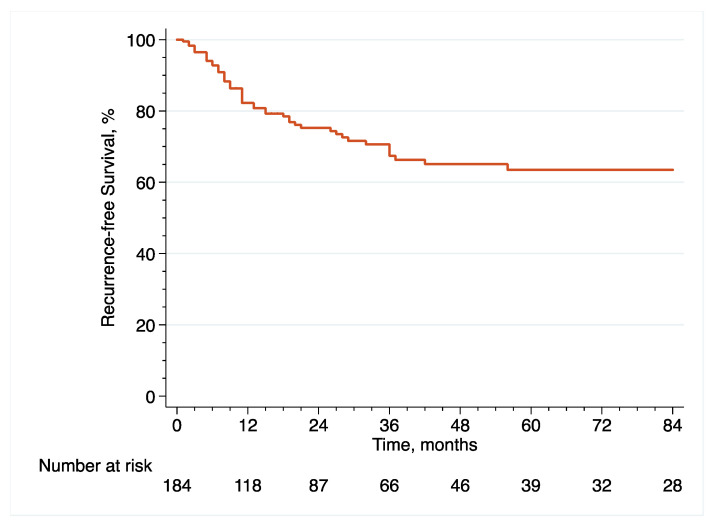
Kaplan–Meier curve that analyses the RFS of included patients treated with eRC for HR-NMIBC.

**Figure 2:**
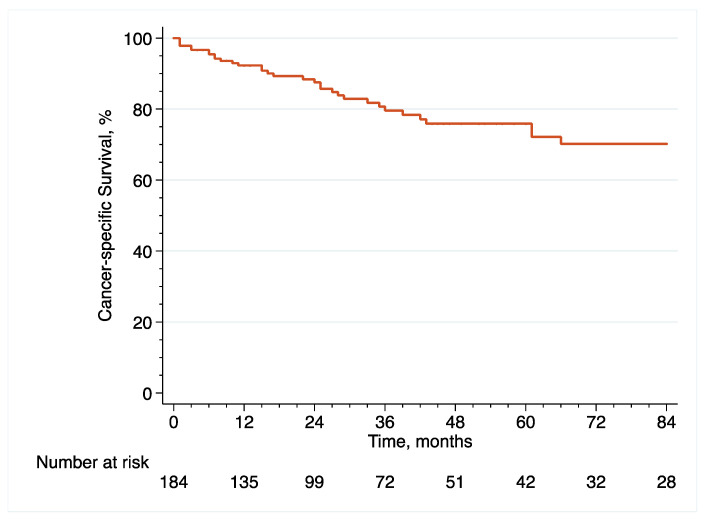
Kaplan–Meier curve that analyses the CSS of included patients treated with eRC for HR-NMIBC.

**Figure 3:**
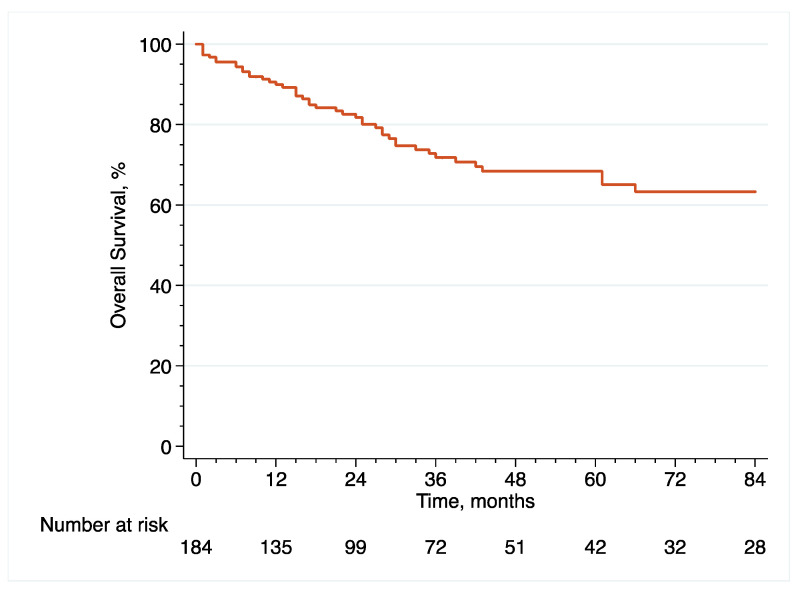
Kaplan–Meier curve that analyses the OS of included patients treated with eRC for HR-NMIBC.

**Figure 6:**
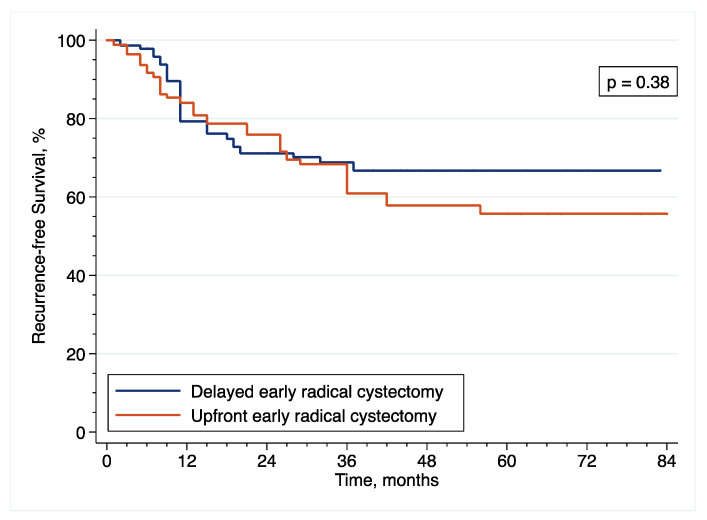
Inverse probability of treatment weighting (IPTW)-adjusted Kaplan–Meier curves that compare the RFS of included patients treated with upfront or delayed eRC for HR-NMIBC.

**Figure 7:**
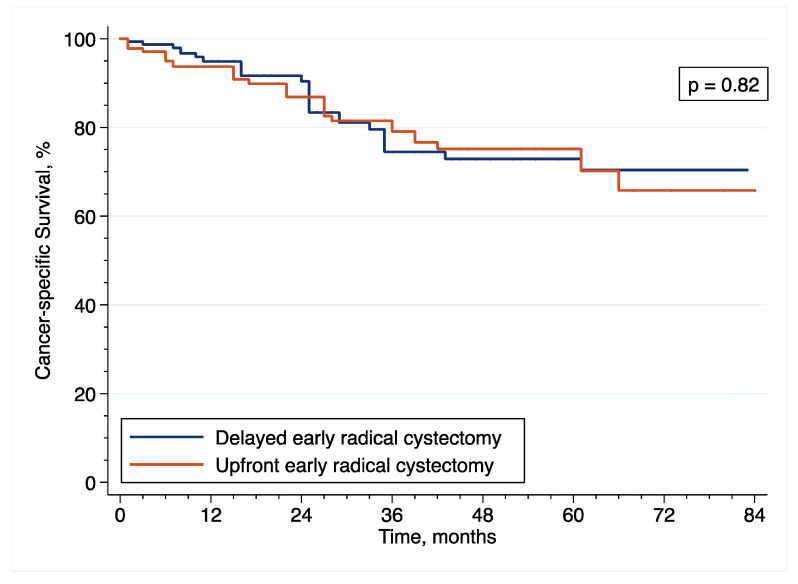
Inverse probability of treatment weighting (IPTW)-adjusted Kaplan–Meier curves that compare the CSS of included patients treated with upfront or delayed eRC for HR-NMIBC.

**Figure 8:**
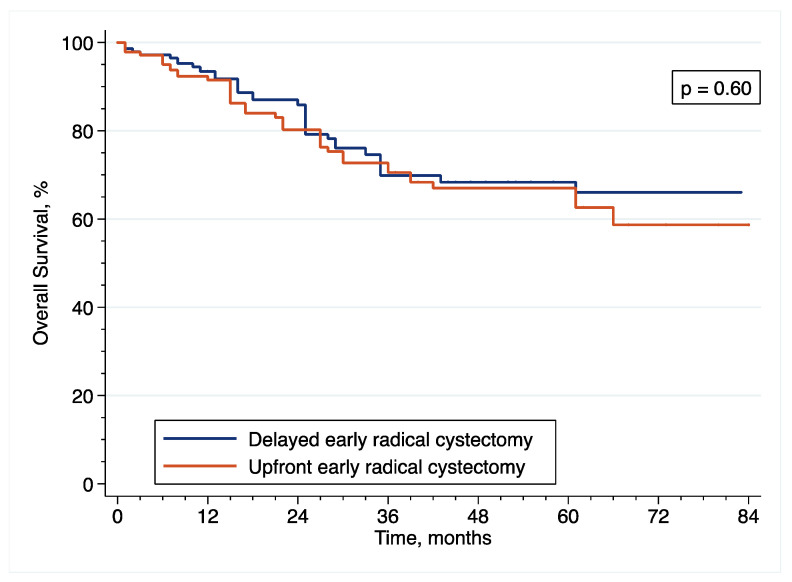
Inverse probability of treatment weighting (IPTW)-adjusted Kaplan–Meier curves that compare the OS of included patients treated with upfront or delayed eRC for HR-NMIBC.
